# Low-Profile, Shoe-Type Ankle–Foot Orthosis with Active Variable Ankle Stiffness via Wire–Fabric Compression Mechanism

**DOI:** 10.3390/biomimetics10080539

**Published:** 2025-08-16

**Authors:** Eunbin Choe, Junyoung Moon, Jaewook Ryu, Seungtae Yang, Alireza Nasirzadeh, Sejin Kong, Youngsuk Choi, Giuk Lee

**Affiliations:** 1 School of Mechanical Engineering, Chung-Ang University, Seoul 06974, Republic of Korea; minnam48@cau.ac.kr (E.C.); wodnr1958@cau.ac.kr (J.R.); alireza@cau.ac.kr (A.N.); 2 HUROTICS Inc., Seoul 06912, Republic of Korea; mjyoung5@hurotics.com (J.M.); 99hilton@hurotics.com (S.Y.); 3 R&D Center, LS Networks Co., Ltd., Seoul 04386, Republic of Korea; sjkong@lsnetworks.com (S.K.); yschoi@lsnetworks.com (Y.C.)

**Keywords:** wearable robots, ankle–foot orthoses, variable stiffness, wire–fabric compression mechanism

## Abstract

Acute ankle sprains frequently lead to chronic ankle instability and muscle atrophy by causing immobilization, which necessitates real-time stiffness modulation for ankle–foot orthoses (AFOs). This paper proposes Active Variable Compression Shoes (AVC-Shoes), an ankle support system inspired by the “heel-lock taping” technique, which employs a wire–fabric compression mechanism to selectively stiffen ankle joints at crucial points in the gait cycle. The experimental results confirmed that AVC-Shoes achieve variable ankle stiffness in all directions, demonstrating dorsiflexion and plantarflexion stiffness ranges of up to 8.3 and 5.9 Nm/rad, respectively. Additionally, preliminary human testing involving three healthy participants revealed that the gastrocnemius muscle activity during the push-off phase in the active compression mode was significantly higher (by 19%) than that in the brace mode. By selectively increasing stiffness at heel strikes, AVC-Shoes represent a promising advancement toward next-generation AFOs capable of stabilizing the ankle while preventing muscle atrophy, which is associated with prolonged brace use.

## 1. Introduction

Acute ankle sprains remain among the most common musculoskeletal injuries, accounting for approximately 15–30% of all sports-related injuries. Moreover, lateral sprains constitute up to 85% of all acute ankle sprains [1,2,3,4,5,6]. Recurrent sprains often result in chronic ankle instability (CAI), characterized by pathological changes in ligamentous and tendinous structures, proprioceptive deficits, and an increased risk of reinjury. Individuals with CAI commonly experience reduced physical performance and diminished health-related quality of life [7]. Consequently, external mechanical interventions designed to enhance ankle stiffness in the frontal plane while preserving sagittal plane mobility have become central to conservative treatment strategies.

Ankle–foot orthoses (AFOs) represent the most widely prescribed external support for ankle injuries. Conventional rigid AFOs, typically thermoformed from lightweight plastics, hold the ankle near neutral, thereby mitigating inversion and eversion excursion and attenuating ligamentous loading [7,8,9,10,11,12,13,14]. While effective at preventing acute buckle events, such constant-stiffness support can restrict dorsiflexion and plantarflexion mobility, alter the spatiotemporal pattern of gait, diminish running and jumping performance, and, with prolonged use, contribute to disuse-related muscle atrophy [15,16,17,18,19,20,21].

Real-time adjustments to ankle stiffness can benefit ankle joint stability and help prevent performance decline. Therefore, researchers have investigated methods to integrate variable-stiffness mechanisms into AFOs. Thalman et al. proposed an AFO featuring a pneumatic actuator, which adjusts the ankle stiffness by controlling the internal air pressure [22,23,24] and demonstrates effective stiffness modulation. However, pneumatic actuation-based approaches have several limitations. For instance, pneumatic actuators typically require considerable space and often necessitate bulky external components, such as compressors, which are incompatible with slim, low-profile designs suitable for daily wear [25]. Additionally, pneumatic systems commonly experience delays due to valve response times, which complicates precise control and potentially causes inaccurate stiffness modulation.

Ham et al. proposed a layer-jamming mechanism to address the limitations of pneumatic actuators in AFOs [26,27]. They integrated the layer-jamming mechanism into the lateral side of the ankle joint and successfully demonstrated its capability to modulate ankle stiffness. However, although their approach enables stiffness adjustment and offers advantages such as portability and a compact, low-profile design, it has certain limitations. Specifically, due to its nature, the layer-jamming mechanism struggles to conform to complex anatomical shapes accurately.

Although variable-stiffness mechanisms have been successfully integrated into AFOs to enhance ankle stability and physical performance, several challenges persist. Addressing these challenges requires developing AFO systems that account for multiple factors beyond stiffness modulation. Based on a review of prior research, we identified five essential design criteria for maximizing the effectiveness of AFOs in improving ankle stability and physical performance:

1. Variable stiffness range;
2. Stiffness magnitude;
3. Geometry dependency;
4. Compactness;
5. Controllability.

**Figure 1:**
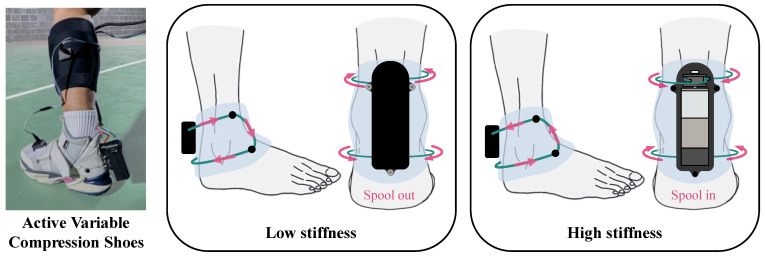
AVC-Shoes: a shoe-integrated AFO with variable stiffness modulation based on a wire–fabric compression mechanism. The light blue shaded area represents the upper part of the boot around the ankle, the green line is the wire, and the black box behind the ankle represents the actuator. The side and rear views of the ankle show how the wire moves when spooled in and out by the actuator.

To simultaneously satisfy these five criteria, we designed an AFO that functions based on a wire–fabric mechanism [28]. Due to the flexibility of the wire and fabric, stiffness can be consistently applied to surfaces with complex shapes. Additionally, the stiffness level can be conveniently modified by adjusting the length of the wire wound onto the motor-driven spool. This motor-driven adjustment also ensures more reliable and easier stiffness control compared to other variable-stiffness mechanisms. Furthermore, the wire–fabric mechanism enables a compact, low-profile system design. Based on this approach, we developed a novel AFO called Active Variable Compression Shoes (AVC-Shoes), shown in Figure 1. A compact, shoe-integrated AFO, AVC-Shoes, utilizes a wire–fabric compression mechanism to apply variable compression force and achieve a geometry-dependent fit, thereby meeting all the aforementioned designed requirements. While prior studies have focused on AFO designs that prevent ankle sprains [29], our design additionally allows compression forces to be applied in all directions. 

Section 2 introduces the design of AVC-Shoes. Section 3 outlines the experimental protocol for evaluating the device’s performance, including preliminary human tests conducted during normal walking. Finally, Section 4 discusses the results, and Section 5 concludes the paper.

## 2. Design of AVC-Shoes

**Figure 2:**
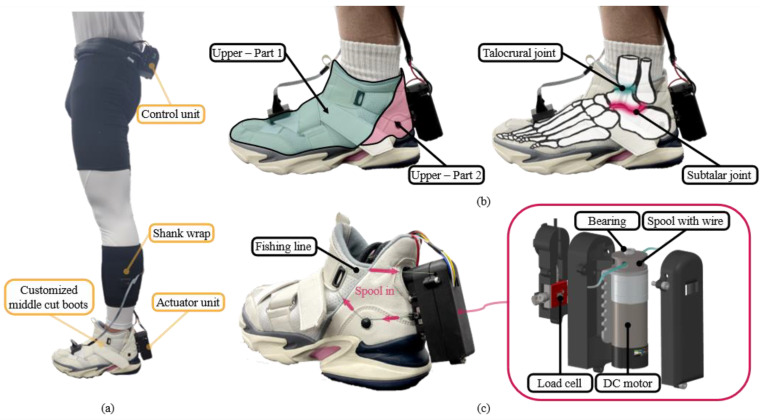
(**a**) Configuration of AVC-Shoes. (**b**) Design of upper part of AVC-Shoes, which is divided into two parts to generate a firm compression force on the talocrural and subtalar joints. (**c**) Design of actuation component. Spooling the fishing line generates a compression force on the ankle joint along the wire path (right inset: complete configuration of the actuator unit).

As shown in Figure 2a, AVC-Shoes comprise a customized middle-cut boot (PRO-SPECS, Seoul, Republic of Korea), a shank wrap, an actuator unit, and a control unit. The total weight of the entire assembly is 916 g; the middle-cut boot, including the actuator unit, weighs 536 g, while the shank wrap and control unit weigh 85 g and 291 g, respectively.

### 2.1. Implementation of Apparel Components: Customized Middle-Cut Boot and Shank Wrap

To increase ankle stability efficiently, the compression force must be delivered to the subtalar and talocrural joints simultaneously [30]. To achieve this, the joints leading to the calcaneus, talus, and tibia must be locked. Thus, we integrate the “heel-lock taping” technique—the most effective taping method to increase ankle stability—into the design of the upper part and wire path of the boot [31]. The upper part is separated into two sections based on the malleolus. This division helps the AFO conform more easily to the shape of the ankle without the upper part wrinkling, which can occur during the winding of the wire; this design facilitates a more efficient transfer of the compression force to the ankle joints. As shown in Figure 2b, the wire path is designed to create a compression force from the calcaneus to the tibia. To provide additional passive stiffness to the talocrural joint, the boot is designed with a middle cut.

### 2.2. Implementation of Actuator Unit

A wire-driven actuator is designed to wind the wire, as depicted in Figure 2c. This actuator comprises cylindrical brushed DC geared motors equipped with encoders (Resolution: 48 CPR; HP 12 V 25D Metal Gearmotors, 47:1, Pololu, Las Vegas, NV, USA) and a 3D printed cylindrical spool (diameter: 21.6 mm; Onyx, Markforged, Natick, MA, USA). A fishing line (ULTRA DYNEEMA, diameter: 0.288 mm, max load: 16.1 kg, GUJU, Japan) serves as the wire. Additionally, a load cell (LSB200; Futek Advanced Sensor Technology Inc., Irvine, CA, USA) is employed to determine the compression threshold during system initialization. The actuators are controlled using commands transmitted from a motor driver (TB9051FTG Single-Brushed DC Motor Driver Carrier, Pololu, USA).

### 2.3. Implementation of Control Unit

A microcontroller unit (ARM Cortex-M7; Teensy 4.1, PJRC, Portland, OR, USA) is utilized to collect sensor data and manage the overall system. Inertial measurement units (IMUs) (BNO055, Adafruit, New York, NY, USA) are attached to the boot to monitor gait cycles. IMU data are collected through I2C communication, while the load cell data are acquired via analog signals. The motor driver is controlled by the microcontroller using pulse-width modulation signals. Power for the actuator and control electronics is provided by a Li-Po battery (11.1 V, 900 mAh 45 C; PT-B900-SP45 3S, Poly-Tronics, Republic of Korea).

## 3. Evaluation of AVC-Shoes

### 3.1. Ankle Stiffness Modulation Performance

#### 3.1.1. Principle of Ankle Stiffness Modulation Using Wire–Fabric Compression Mechanism

**Figure 3:**
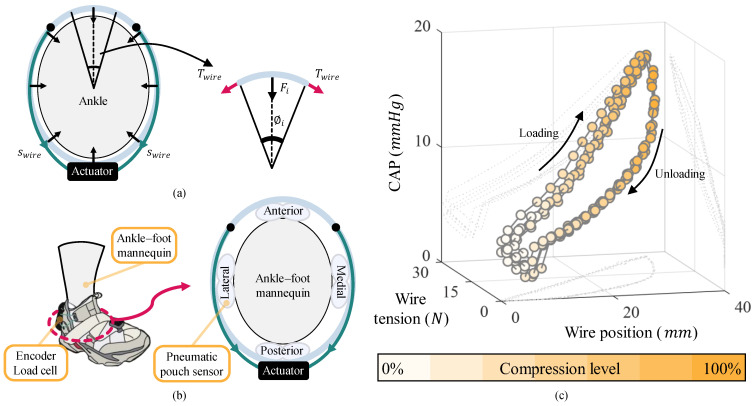
Concept of wire–fabric compression mechanism. (**a**) Top view of the ankle, showing how a compression force is applied to the ankle by the tension in the wire. (**b**) Mannequin-based testbed and data flow for validation of wire–fabric compression mechanism. (**c**) Data measured from the testbed, which include changes in wire tension and circumferential ankle pressure based on the wire position. The yellow gradient represents the compression level based on the wire position.

Figure 3 explains the wire–fabric compression mechanism implemented in AVC-Shoes, which generates compression forces around the ankle. Figure 3a demonstrates that the fabric connected to the wire path stretches as the wire is wound. The displacement of the wound-up wire ($s_{wire}$) increases the tension in the wire ($T_{wire}$), which is proportional to the overall passive stiffness of AVC-Shoes ($k_{shoe}$). Additionally, $k_{shoe}$ is a combination of the stiffness of the wire and the upper-part fabric.

The wire tension ($T_{wire}$) generates an infinitesimal compression force ($F_{i}$) on the ankle:

(1) $$T_{wire}=\;k_{shoe}*s_{wire}.$$

This force is proportional to the contact angle ($\phi_{i}$) [32]:

(2) $$F_{i}=\;2T_{wire}*\sin(\frac{\phi_{i}}{2})\;\approx T_{wire}*\phi_{i.}$$

To verify the relationship between the wire position, wire tension, and compression forces, a mannequin-based testbed was constructed, as shown in Figure 3b. The wire position was calculated using the motor encoder data, and the wire tension was measured using a load cell connected to the wire. The compression forces were indirectly measured in terms of the circumferential ankle pressure (CAP), which represents the average pressure recorded by pneumatic pouch sensors attached to the anterior, posterior, lateral, and medial sides of the ankle–foot mannequin.

Before the measurements were performed, the AVC-Shoes entered the initialization stage to identify and set the maximum amount that could be wound on the spool. The motor position at which the load-cell measurement did not increase further when more wire was wound onto the spool was designated as the maximum motor position ($P_{max}$). Conversely, the point at which the load-cell measurement became zero while unwinding the spool was set as the base motor position ($P_{0}$).

The tension in the wire was measured using a load cell while the wire was wound and unwound at a constant speed of 0.01 m/s. The data were recorded across six cycles of operation.

Figure 3c shows the variation in the wire tension and CAP with the wire position. The seven levels of yellow saturation on the scale represent seven distinct compression levels, ranging from $P_{0}$ (0%) to $P_{max}$ (100%). Thus, Figure 3c demonstrates that AVC-Shoes can generate variable compression forces through the adjustment of the motor position. Specifically, as the wire tension increased from 0 N to 28 N, the CAP increased from 2.3 to 18.2 mmHg.

#### 3.1.2. Evaluation of Ankle Stiffness Modulation

**Figure 4:**
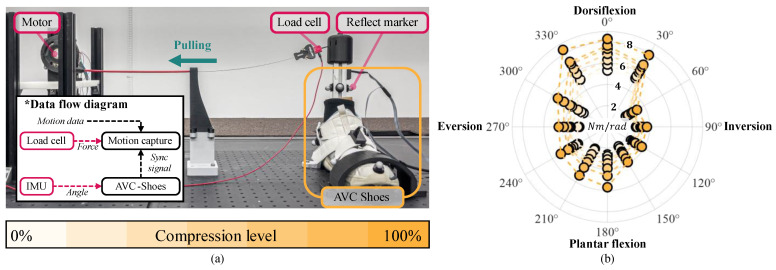
(**a**) Configuration of hardware and data flow on testbed for validating stiffness modulation performance of AVC-Shoes. (**b**) Wind rose graph showing the stiffness variation in AVC-Shoes, with the peak stiffness shown across the seven compression levels.

To evaluate the ankle stiffness modulation capability of AVC-Shoes, we established the test setup depicted in Figure 4a. The AVC-Shoe unit was mounted on an ankle–foot mannequin that was fixed onto a base plate. To capture the ankle joint angles precisely, reflective markers were attached to both the AVC-Shoes and the mannequin. The movements of these markers were recorded by an eight-camera motion capture system (Arqus A5, Qualisys, Sweden) operating at a sampling frequency of 500 Hz. The captured motion data were smoothed using the built-in filtering function provided by the Qualisys Track Manager 2024.1 software program (Qualisys, GÖteborg, Sweden).

The ankle joint was actuated by pulling a Bowden cable connected to a motor. A load cell (LSB200; Futek Advanced Sensor Technology Inc., Irvine, CA, USA) was placed between the Bowden cable and the mannequin to record the pulling force applied to the ankle. The load-cell signals were collected as analog data using the aforementioned motion capture system at a sampling rate of 2000 Hz, before being filtered using a low-pass filter (cutoff frequency: 10 Hz) in MATLAB R2023b (MathWorks, USA). The motor operation during the experiments was controlled using a real-time position control system implemented in the Elmos Studio II software tool (Elmo Motion Control, Herzliya, Israel) and a motor driver (Gold Solo Twitter 10/100, Elmo Motion Control, Israel).

An experiment was performed to assess the variable-stiffness characteristics of AVC-Shoes at different compression levels. The compression provided by AVC-Shoes was categorized into seven distinct levels ($P_{0}-P_{max}$). To acquire data reflecting realistic walking conditions, the ankle deflection angle and angular velocity were fixed at 30° and 100°/s, respectively [33]. The varying stiffness was measured at 30° intervals around the ankle, starting from the anterior direction, to assess the stiffness in all directions; the same measurements were also conducted without the shoe. To calculate only the stiffness provided by AVC-Shoes, the stiffness under the no-shoe condition was subtracted from the stiffness measured while the shoe was worn.

Figure 4b presents the measured variations in stiffness. Under dorsiflexion and plantar flexion, the maximum stiffness values were 8.3 and 5.9 Nm/rad, respectively, with the corresponding variation ranges being 3.4 and 3.7 Nm/rad, respectively. Conversely, under inversion and eversion, the maximum stiffness values were 3.9 and 5.2 Nm/rad, respectively, while the variation ranges were 1.0 and 1.9 Nm/rad, respectively.

### 3.2. Human Test Under Walking Conditions

#### 3.2.1. Experimental Protocol

**Figure 5:**
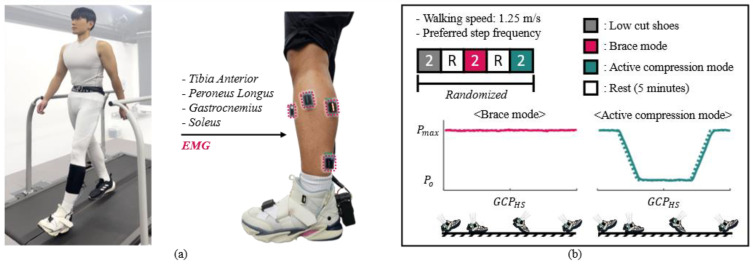
Experimental setup and protocol for human test under walking conditions. (**a**) Photograph of experimental setup. (**b**) Experimental protocol.

We conducted a preliminary test to verify the human response to the variable ankle stiffness provided by AVC-Shoes during walking. The experimental protocol was approved by the Chung-ang University Institutional Review Board (1041078-202107-HR-214-0). Three healthy male participants with no musculoskeletal injuries of the ankle joint (mean ± standard deviation; age, 25 years; height, 1.74 ± 0.03 m; weight, 73.7 ± 13.58 kg) were recruited. As shown in Figure 5a, the participants wore an AVC-Shoe on their left foot and walked on an instrumented treadmill FIT5 (Bertec, Columbus, OH, USA). We also attached a wireless surface electromyography (EMG) system (Trigno system, Delsys, Natick, MA, USA) to each participant’s left shank. The EMG data were measured on four muscles: tibialis anterior, peroneus longus, lateral gastrocnemius, and soleus.

We measured the human response from the participants under three conditions. Under the first condition, the participants wore general-purpose low-cut athletic shoes (Alphalava, Adidas, Germany). Under the second condition, they wore AVC-Shoes that always provided the maximum motor position ($P_{max}$) for the ankle (brace mode). Under the third condition, they wore AVC-Shoes that applied the maximum compression force only during the heel-strike event (active compression mode). In the active compression mode, the compression force is applied only at the point of initial foot contact, when the ankle is most unstable; no compression force is applied to the ankle during the single-support phase, when the ankle is relatively stable [34]. The heel-strike-based gait cycle percentage (GCP) was calculated using the sagittal angular velocity recorded by the IMUs placed on the shoes [35]. The experimental protocol is illustrated in Figure 5b. The participants walked on a treadmill at a speed of 1.25 m/s for 2 min per trial. The trials for each of the three conditions were conducted in a random order. The participants were allowed 5 min of rest after each trial to enable sufficient recovery from fatigue.

The EMG signals were acquired at 2000 Hz and were passed through three types of filters: first, a band-pass filter (20–460 Hz, fourth-order Butterworth) was applied; then, the signal was rectified; finally, it was smoothed using a low-pass filter (6 Hz, fourth-order Butterworth). The processed EMG data for each muscle were normalized by the relative voluntary contraction (RVC) recorded during the low-cut-shoe trial. The data were split into distinct gait cycles based on the heel strikes of the left side leg and then averaged for each muscle across the shoe types and participants.

#### 3.2.2. Human Experiment Result

**Figure 6:**
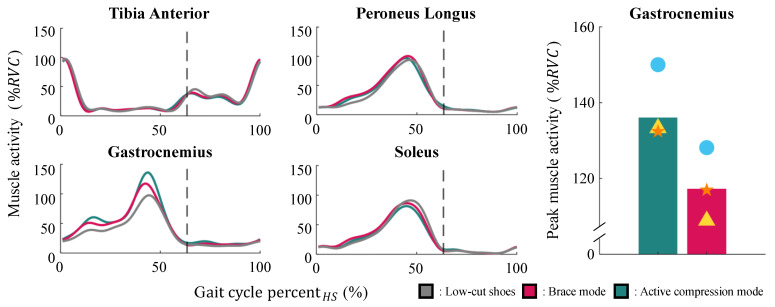
Human experiment results. The left-side graph shows the activity levels of the four muscles on the shank in terms of the GCP. The dashed line represents the toe-off event. The right-side bar graph shows the differences in the average peak activity of the gastrocnemius muscle between brace mode (red bar) and active compression mode (green bar) for each participant. The circle, triangle, and star symbols represent the three participants.

Figure 6 shows the muscle activity in terms of the average GCP for the three subjects. The active compression mode significantly increased the activity of the gastrocnemius muscle in the push-off phase. The peak activity of this muscle in the active compression mode was 19% higher than that in the brace mode and 36% higher than that during the low-cut-shoe trial. The right side of Figure 6 shows the extent to which the activity of the gastrocnemius muscle increased for each subject. No change in activity was observed for the remaining muscles when the subjects wore AVC-Shoes instead of the low-cut shoes.

## 4. Discussion

In this study, we developed a variable-stiffness AFO based on a wire–fabric mechanism capable of adjusting ankle joint stiffness by applying compressive forces in all directions. The stiffness in the dorsiflexion direction was found to be notably higher, ranging from approximately 1.8 times ($P_{max}$, at 100%) to 7.6 times ($P_{0}$, at 0%) higher than in the other directions. This directional stiffness disparity primarily results from the design of the upper part of the AVC-Shoes, which is divided around the malleolus. Specifically, the higher stiffness observed in the dorsiflexion direction can be attributed to additional passive stiffness obtained from the shoe’s high collar. Conversely, in the mediolateral directions, the stiffness relies solely on the compression generated by the wire–fabric mechanism due to the separation of the upper segment. This behavior resembles the problem observed in loosely designed heel cups intended for easy fitting and removal. To overcome this limitation, future designs could utilize different materials or arrangements in which the two upper segments overlap rather than remaining independent.

Additionally, we analyzed how the variable ankle stiffness provided by AVC-Shoes influences human response during walking. The activity of the gastrocnemius muscle during the push-off phase was higher in the active compression mode than in the brace mode. AFOs can lower the activity of the gastrocnemius muscle as they reduce the passive tension in the Achilles tendon by restricting the elongation of the tendon during dorsiflexion [21,36,37]. The active compression mode allows dorsiflexion during the stance, thus potentially increasing the activity of the gastrocnemius muscle. Fröberg et al. reported that AFOs that allow dorsiflexion produce minimal reductions in muscle activity compared to conventional AFOs and can prevent muscle atrophy [21]. Thus, AVC-Shoes can be considered to prevent muscle atrophy because they increase ankle stiffness only during heel strike.

Contrary to our hypothesis, however, the activity of each participant’s gastrocnemius muscle while wearing the low-cut shoes was lower than while wearing the AFO. This discrepancy is attributed to the greater weight of AVC-Shoes (536 g) compared to the low-cut shoes (245 g) [38]. The difference in the flexibility of the shoe sole could also have contributed to the difference in muscle activity [39]. To determine how the muscle activity depends on the AFO stiffness, experiments with shoe stiffness and weight as control variables must be conducted.

No change in the activity of the soleus muscle was observed between the three experience conditions. We hypothesized that the soleus muscle’s activity would be similar to that of the gastrocnemius muscle because the soleus muscle is also affected by the passive tension in the Achilles tendon. Since lower-leg muscle activity is influenced by joint motion, the unexpected results can be ascribed to factors that we did not consider [40]. Specifically, the upper part of AVC-Shoes actively moves during walking, which may have affected the accuracy of the ankle joint marker data. Thus, we were unable to obtain reliable joint motion data using the motion capture system. Since this conventional system could not provide reliable data, we are planning to conduct an experiment using a dual fluoroscopic imaging system to obtain more accurate joint motion data.

Previous studies have suggested that increased ankle stability reduces the activity of the tibia anterior and peroneus longus muscles [21,41]. However, the activity of these muscles did not change when the participants switched to the AFO. This observation is attributed to the low compression force provided by AVC-Shoes, which was not strong enough to increase ankle stability. The maximum compression force provided by AVC-Shoes was 18.2 mmHg (based on the CAP), which is equivalent to the level of compression stockings. With compression forces of this level, completely restricting ankle movement with an AFO is difficult. Nasirzadeh et al. developed passive dial-adjusted compression shoes that can apply a compression force of over 60 mmHg [42]. However, even those shoes could not restrict ankle joint movement completely, thus being unable to reduce muscle activity. In future studies, we will aim to enhance ankle stability by improving the shoe design and actuator performance to apply stronger compression forces on the ankle. Moreover, we acknowledge that the results of our human tests have limitations, as these tests involved only three healthy male participants. To validate the effect of the device more rigorously, experiments involving more subjects, including female participants, will need to be conducted.

## 5. Conclusions

This study introduced AVC-Shoes, a novel shoe-integrated AFO that uses a wire–fabric mechanism to modulate ankle stiffness in every direction. Compared to existing devices, AVC-Shoes are lighter, more compact, and more comfortable, while their motor-based design also makes them easier to control. Because AVC-Shoes can adjust the ankle stiffness according to the user’s gait cycle, they deliver the optimal amount of support at the appropriate time. To the best of our knowledge, no other mobile system can actively tune ankle stiffness in all directions. A preliminary human study also suggests that the device can help prevent muscle atrophy by allowing the ankle to move more naturally.

However, several improvements are still needed. First, the actuator must be upgraded to ensure that it can tighten faster and with greater force. Second, we plan to redesign the upper part to mitigate the stiffness differences between different movement directions. Finally, instead of single-leg tests, we will conduct experiments involving bilateral use and will assess the performance of the AFO under more diverse walking conditions and with various assistance strategies.

## Data Availability

The data are contained within the article and Supplementary Materials.

## Supplementary Materials

The following supporting information can be downloaded at https://www.mdpi.com/article/10.3390/biomimetics10080539/s1, Video S1: Active Variable Compression Shoes: Design and Evaluation of Ankle–Foot Orthoses for Variable Ankle Stiffness via Wire–Fabric Compression Mechanism.
